# Epidemiology of head injuries and helmet use among motorcycle crash injury: a quantitative analysis from a local hospital in Western Kenya

**DOI:** 10.11604/pamj.2018.31.70.16988

**Published:** 2018-10-02

**Authors:** Peter Kiteywo Sisimwo, Geoffrey Mose Onchiri

**Affiliations:** 1 Kenyatta National Referral Hospital, Nairobi, Kenya

**Keywords:** Epidemiology, head injury, helmet use, motorcycle crash injury, Kenya

## Abstract

**Introduction:**

Injury from motorcycle is a considerable cause of deaths and disability in the world. It is becoming one of the most serious public health problems, not only in developed countries but more in low and middle-income countries.

**Methods:**

Descriptive cross sectional study for patients who sustained head injuries related to motorcycle crashes between March 2017 and March 2018. Participant bio data, injury history and neurological examination findings were collected using pretested interviewer administered questionnaires. Frequencies, Mean (SD) and chi-square was employed in the analysis. Results were considered significant at p<0.05.

**Results:**

A total of 341 motorcycle crash injury patients participated in the study. One hundred and forty three (42%), sustained head injuries while 198 (58%) sustained other types of injury. In regard to safety helmets, 94 (28%) wore helmets at the time of crash. One hundred and forty three (42%), of the respondents without helmets at the time of crash sustained head injuries. Riders and passengers who wore helmets at the time of motorcycle crash, suffered less frequent head injuries compared to those who did not wear helmets and this was statistically significant (χ2=55.78, P<0.00). Non use of safety helmet during crash was associated with sustaining mild to severe head injury. Most of the crashes 165 (48.3%), occurred as a result of collision between motorcycles versus vehicle. Majority of the crashes occurred in the afternoon hours 174 (51%). The days of the week recording the highest number of injuries were Friday (16.1%) and Monday (15.8%). The day of Involvement in motorcycle crash during the week was not significantly associated with head injury (χ2=13.103, p=0.785).

**Conclusion:**

Majority of motorcycle crash injury victims sustained head injury. Few of the victims used safety helmets at the time of the motorcycle crash. Use of helmets was protective of sustaining mild to severe head injuries among crash injury victims.

## Introduction

Road traffic injuries are a worldwide disaster. The World Health Organization (WHO) estimates that about 1.2 million die and 50 million are injured yearly [1]. Unfortunately, a disproportionate burden of this injury is currently and will continue to be borne by low income and middle income countries [1, 2]. Some of this increase has been fueled largely by the escalating use of motorcycles for commercial transportation of commuters, goods, and services [3, 4]. Motorcycles are the most dangerous type of motor vehicles to drive, accounting for higher rates of crashes and fatalities compared to passenger cars per miles driven [5]. Injury from motorcycle is a considerable cause of deaths and disability in the world. It is becoming one of the most serious public health problems, not only in developed countries but most especially in low and middle-income countries. Motorcycle is used purposely for transportation because it is cheap and fast access to areas not pliable by motor vehicles [5]. Motorcycle helmets are effective in reducing head and neck injuries and deaths from motorcycle crashes. Studies indicate that wearing helmets reduces fatalities by more than 25% [6]. The National Highway Traffic Safety Administration (NHTSA) estimates that wearing helmets reduces motorcyclists overall risk of death in a crash by 29% and the risk of brain injury by 67% [7].

Helmets usually made of a rigid fiberglass or plastic shell, a foam liner, and a chinstrap, have been the principal countermeasure for preventing or reducing head injuries from motorcycle crashes. Based on police reports, helmets reduced the risk of motorcycle deaths by 29% during 1972-1987 [7]. In East Africa, motorcycle related crashes contribute 21- 58.8% of the road traffic crashes with head injury being common [8]. Most of the crash victims require surgical intervention and survivors tend to have chronic disabilities [8]. Despite the burden of the problem in Kenya, motorcycle injuries have not received the attention they deserve. There is need for more literature on these injuries that will inform stakeholders on the magnitude of the problem. Majority of motorcycle injuries are preventable, a clearer understanding of the magnitude, contributing factors and injury patterns is essential for establishment of prevention strategies as well as treatment protocols. This study documents the epidemiology of head injuries and helmet use among motorcycle crash victims seen at Kitale County referral hospital.

## Methods

Following ethical approval from Kenya Medical Research Institute Review Board, a descriptive cross sectional study was conducted in 2017 at the Accidents and Emergency Department of Kitale Country Referral Hospital. Kitale town is located at high Agricultural potential area of Trans-Nzoia County with an estimated population of 200,000. On average, 10 to 20 motorcycle crash victims are attended to daily. Trauma patients are first resuscitated and managed at the A&E Department according to the Advanced Trauma Life Support (ATLS) principles and then admitted to the admitting surgical firm. All victims of motorcycle crash injuries presenting at the accident and emergency department were eligible for the study. Consecutive consenting participants were prospectively enrolled. At presentation, patients or their conveyers were interviewed by research assistants according to the details provided on a pro-forma. Participant bio data, injury history and neurological examination findings were collected using pretested interviewer administered questionnaires. The personal status during the crash was identified whether he/she was the rider, passenger and pedestrian. Head injury severity was measured using the Glasgow Comma Scale.

Motorcycle crash victims who came unconscious were also enrolled in this study after consent was obtained from their relative or from themselves after gaining conscious either in ICU or in the ward. Two trained research assistant were stationed in the accident and emergency department who interviewed patients and did data entry. Diagnosis was reached through clinical history, examination and radiological investigations. Topographic locations of injuries were then entered in the structured questionnaire. Information about diagnosis, registration number was retrieved from patient's file and admission register books. Data cleaning and validation was performed using SPSS version 20. Descriptive statistics such as mean, standard deviation, range and frequency proportions was used to summarize the data. Pearson's chi square was used to test for the significance of association between dependent variable and independent variables. The level of statistical significance was set at P <0.05.

## Results

A total of 341 motorcycle related crash injury patients participated in the study. Out of the 341 patients recruited, 143 (42%) of them sustained head injuries while 198 (58%) sustained other types of injury. In this study, 118 (34.6%) of the head injury patients were in the 20-29 age bracket, followed by those in the 30-39 (31.7%), 10-19 (15.5%), 40-49 (8.5%), 50-59 (5.6%) and >60 (41%) respectively. The combined mean age in years was 30.98 (SD 12.905). For female participants the mean age was 31.43 (SD 12.682) and 31.41 (SD 11.489) for males as illustrated in (Table 1). Most respondents 213 (62.5%) had attained primary school level of education. One hundred and nineteen (34.9%) had attained secondary education and 9 (2.6%) tertiary level of education. Most respondents were motorcycle riders 168 (49.3%), fifty five (16.1%) were students, businessmen/women 39 (11.4%), farmers 36 (10.6%), drivers 4 (1.2%), housewives 19 (5.6%) and others 20 (5.9%). One hundred and ninety eight (58.1%) of the respondents were married, 141 (41.3%) were single, 1 (0.3%) divorced and 1 (0.3%) widowed. In regard to motorcycle safety helmets, only 94 (28%) wore helmets at the time of motorcycle crash. One hundred and forty three (42%), of the respondents without helmets at the time of motorcycle crash sustained head injuries. Two hundred and forty seven (72%) of respondents did not wear safety helmets at the time of motorcycle crash. Use of helmet was protective of head injury during motorcycle crash and was statistically significant (χ2=55.78, P<0.00). Conversely non use of safety helmet during motorcycle crash was associated with sustaining mild to severe head injury.

**Table 1:** Socio demographic characteristics of the study population, Kitale, Kenya 2017-2018 (n=341)

characteristic	Female (n=74) No (%)	Male (n=267) No (%)	Total (n=341) No (%)
**Age in years (Mean , SD)**	31.43 (12.682)	31.41 (11.489)	30.98 (12.905)
**Age Group (Years)**			
10-19	11(14.9)	42(15.7)	53(15.5)
20-29	26(35.1)	92(34.5)	118(34.6)
30-39	23(31.1)	85(31.8)	108(31.7)
40-49	6(8.1)	23(8.6)	29(8.5)
50-59	4(5.4)	15(5.6)	19(5.6)
>60	4(5.4)	10(3.8)	14(4.1)
**Occupation**			
Business	8(10.8)	31(11.6)	39(11.4)
Motorcycle rider	36(48.6)	132(49.4)	168(49.3)
Driver	1(1.4)	3(1.1)	4(1.2)
Student	12(16.2)	43(16.1)	55(16.1)
Farmer	8(10.8)	28(10.5)	36(10.6)
Housewife	4(5.4)	15(5.6)	19(5.6)
Others	4(5.4)	16(5.9)	20(5.9)
**Education**			
Primary	46(62.2)	167(62.5)	213(62.5)
Secondary	26(35.1)	93(34.8)	119(34.9)
Tertiary	2(2.7)	7(2.6)	9(2.6)
**Marital status**			
Single	31(41.9)	110(41.2)	141(41.3)
Married	43(58.1)	55(58.1)	198(58.1)
Divorced	0(0)	1(0.4)	1(0.3)
Widowed	1(1.4)	0(0)	1(0.3)

**Figure 1:**
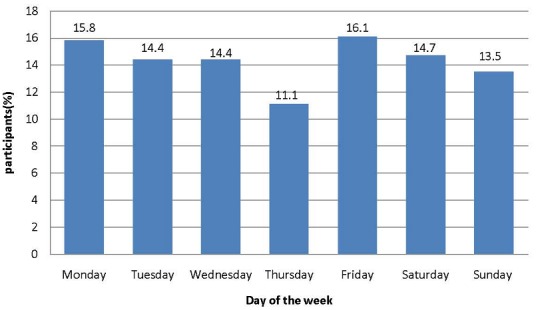
Day of the week study participants were involved in motorcycle crash injury, Kitale, Kenya, 2017-2018 (n=341)

Among the road user category motorcycle riders were the most affected 168 (49%), followed by passengers at 120 (35%). Pedestrians who sustained injuries were 53 (16%). Being a motorcycle rider was highly associated with sustaining head injury and was statistically significant (χ2=80.658, p<0.00) (Table 2). Most of the motorcycle crashes 165 (48.3%) occurred as a result of collision between motorcycles versus vehicle. Motorcycles collision versus motorcycle comprised of 77 (22.6%). Collision between motorcycles and pedestrians accounted for 58 (17%). Motorcycle versus bicycle collision comprised of 32 (9.4%) of the cases. Motorcycle versus animal collision was reported in 5 (1.5%) of the cases while motorcycle collision with poles and tress accounted for 2 (0.6%) as illustrated in (Table 3). Majority of the motorcycle crashes occurred in the afternoon hours 174 (51%). In the morning hours 125 (36.7%) of the crashes took place while 35 (10.3%) of the motorcycle crashes occurred in the evening as illustrated in (Table 3). As regards to the day of the week that motorcycle crashes occurred, majority (71.8%) of the victims sustained head injuries between Monday and Friday. The days of the week recording the highest number of injuries were Friday (16.1%) and Monday (15.8%) as illustrated in (Figure 1). The day of Involvement in motorcycle crash during the week was not found to be significantly associated with head injury (χ2=13.103, p=0.785).

**Table 2:** prevalence of helmet use among the study population, Kitale, Kenya, 2017-2018 (n=341)

Characteristic	Motorcycle helmet use		Total N (%)
	Yes N (%)	No N (%)	
Head injury	39 (27)	104 (73)	143 (42)
No head injury	55 (28)	143 (72)	198 (58)
Total	94 (28)	247 (72)	341 (100)

c^2^=55.76, p<0.00

**Table 3:** Relationship between head injury and various patient characteristic, Kitale, Kenya (n=341)

Characteristic	Head injury (n=143) No (%)	No head injury (n=198) No (%)	Total (n=341) No (%)	P value
**Road user category**				
Rider	71(50)	97(49)	168(49)	χ2=80.658, p=0.00
Passenger	50(35)	70(35)	120(35)	
Pedestrian	22(15)	31(16)	53(16)	
**Mechanism of motorcycle crash injury**				
Motorcycle vs. motorcycle	32(22.4)	45(22.7)	77(22.6)	χ2=97.969, p= 0.00
Motorcycle vs. vehicle	70(49)	95(48)	165(48.3)	
Motorcycle vs. pedestrian	24(16.8)	34(17.2)	58(17)	
Motorcycle vs. animal	2(1.4)	3(1.5)	5(1.5)	
Motorcycle vs. bicycle	13(9)	19(9.6)	32(9.4)	
Motorcycle vs. pole/tree	1(0.7)	1(0.5)	2(0.6)	
Lone vs. motorcycle	1(0.7)	1(0.5)	2(0.6)
**Time of the day crash injury occurred**				
Morning (7AM-11.59AM)	52(36.4)	73(36.9)	125(36.7)	χ2=96.866, p= 0.875
Afternoon (12pm-5.59pm)	73(51)	101(51)	174(51)	
Evening (6pm-11.59pm)	15(10.5)	20(10.1)	35(10.3)	
Early morning (12am-6.59am)	3(2.1)	4(2)	7(2)	

## Discussion

This study highlights the epidemiology of head injuries and helmet use among motorcycle crash injury victims. The study revealed low prevalence of helmet use and a significant occurrence of head injury. Given the rapid increase of motorcycles on Kenyan roads, there is an urgent need for sustained efforts at reducing the risk of head injuries and their consequences among motorcyclists. Official data by the Kenya National Bureau of Statistics shows new motorcycle registries for the 10-month (January 2016- October 2016) period rose to 159,260 units compared to 85,073 units registered during the same period in 2016 [9]. The majority of motorcycle crash injury victims were of the age between 20 and 29 years. This is similar to other studies where the majority of patients involved in motorcycle crash were aged between 20 and 29 years [10]. In contrary one study done in Lagos Nigeria reported the majority of motorcycle crash injury victims to be the age of 31 - 40 years [11]. The reason why the youth are involved in commercial motorcycle could be explained by the fact that at this age group majority are engaged in productive activities predisposing to risks of being involved in road traffic crashes.

In this study 62.5% of victims had primary level of education. This perceived low level of literacy could be due to their inability to appreciate safety messages and codes. This supports another study in which motorcyclist with higher level of education were found to practice safety codes more regularly [12]. In addition, illiterate riders may not be able to interpret road signs thus contributing to road crashes. Among the road user category, motorcycle riders comprised (49%), followed by the passengers (35%) and then the pedestrians (16%). Similar observation was made in Lagos Nigeria where they looked at the patterns of injuries in fatal motorcycle crashes [11]. The increased likelihood of motorcycle occupants to sustain injuries in the event of a crash is because there is no protection over the inherently unstable motorcycle. The high susceptibility of pedestrians to motorcycle crash injuries seen in this study may be because there are no designated pedestrian walk ways in many roads and as such pedestrians walk along the road thereby exposing them to impact from a motorcycle in the event of a crash.

Introduction of mandatory helmet use laws has been shown to be effective in reducing head injuries in a number of countries [13]. In Kenya, the Traffic Amendment Bill that included mandatory helmet use for all motorcyclists, drivers and passengers, was passed in 2009 and penalties were increased in the Traffic Amendment Act in 2012 [14]. While motorcycle helmets have been consistently found to be effective in reducing the risk of death and head injury among motorcyclists in crashes [15], their use in low and middle income countries such as Kenya has historically been low [16]. This study highlights the low prevalence of helmet use in Kenya and the urgent need for Sustained efforts to improve this burden and reduce the risk of head injuries and their consequences among motorcyclists. This is especially true given the rapid increase in the Number of motorcycles on Kenyan roads in recent years. Similar observation of low prevalence of helmet use among motorcycle riders in Thika and Naivasha Kenya was found to be 35.12% and 37.42%, respectively [17]. In this study 28% of the victims reported wearing crash helmets at the time of motorcycle crash. In Kampala Uganda the reported prevalence of helmet use was 18.6% [18]. This low level of helmet use presents an opportunity to further increase both awareness as well as enforcement of the legislation to improve helmet use and ultimately head injury outcomes among motorcyclists.

The commonest cause of motorcycle crash was collision with a vehicle. Vehicles have been reported to contribute majority of motorcycle crashes mainly due to their inability to detect or recognize them in traffic [3]. This shows that probably co-operation, awareness and concern for others and good riding and driving habits are essentials among all road users. Similar observation was made in Kampala Uganda where half of the crashes occurred between a motorcycle and other vehicles [18]. The findings of this study revealed that 72% of the victims sustained head injuries between Monday and Friday. The days of the week recording highest number of injuries were Friday at 16% and Monday at 15.8%. The study also revealed that 51% of the motorcycle crashes occurred in the afternoon hours. The day of involvement in motorcycle crash was not significantly associated with sustaining head injuries. According to the world report on Road Traffic injury prevention, the day of the week is an important factor associated with road traffic crashes [1]. High frequencies of crashes on Monday can be explained by the fact that it is the 1st day of the week and most travelers are reporting to work after spending weekends up country. Most events occur over the weekends and therefore many people travel on Friday. Many urban centers have open air markets on Friday thereby explaining high levels of traffic congestion. More crashes occurred in the afternoon and evening which can be attributed to rider fatigue, traffic rush hours and poor visibility.

## Conclusion

Majority of motorcycle crash injury victims seen at the county referral hospital sustained head injury. Few of the crash injury victims used safety helmets at the time of the motorcycle crash. Use of motorcycle helmets was protective of sustaining mild to severe head injuries among crash injury victims.

### What is known about this topic?

- Motorcycle riders are at greatest risk of suffering severe head injury;
- Low use of protective gear (helmets) by motorcycle users.

### What this study adds

- Majority of the motorcycle crash victims sustaining head injury were in the 20-29 age brackets;
- This study highlights the low prevalence of helmet use among motorcycle riders and their passengers;
- Majority of the motorcycle crashes occurred during the weekdays and in the afternoon/evening hours of the day.

## Competing interests

The authors declare no competing interests.
